# Targeting constitutively-activated STAT3 in hypoxic ovarian cancer, using a novel STAT3 inhibitor

**DOI:** 10.18632/oncoscience.26

**Published:** 2014-03-31

**Authors:** Georgia A. McCann, Shan Naidu, Kellie S. Rath, Hemant K. Bid, Brent J. Tierney, Adrian Suarez, Saradhadevi Varadharaj, Jianying Zhang, Kálmán Hideg, Peter Houghton, Periannan Kuppusamy, David E. Cohn, Karuppaiyah Selvendiran

**Affiliations:** ^1^ Department of Obstetrics and Gynecology, Division of Gynecologic Oncology, Comprehensive Cancer Center, The Ohio State University Wexner Medical Center, Columbus, OH; ^2^ Nationwide Children's Hospital, Columbus, OH; ^3^ Department of Pathology, Divisions of Gynecological Pathology and Cytopathology, The Ohio State University Wexner Medical Center, Columbus, OH; ^4^ Department of Internal Medicine, The Ohio State University Wexner Medical Center, Columbus, OH; ^5^ Comprehensive Cancer Center, The Ohio State University Wexner Medical Center, Columbus, OH; ^6^ Institute of Organic and Medicinal Chemistry, University of Pécs, Pécs, Hungary; ^7^ Department of Radiology, The Dartmouth Medical School, Hanover, NH

**Keywords:** Hypoxia, STAT3, Ovarian Cancer, HO-3867, Chemo-resistance

## Abstract

Tumor hypoxia, a feature of many solid tumors including ovarian cancer, is associated with resistance to therapies. We previously demonstrated that hypoxic exposure results in increased expression of phosphorylated signal transducer and activator of transcription 3 (pSTAT3). We hypothesized the activation of STAT3 could lead to chemotherapeutic resistance in ovarian cancer cells in hypoxic conditions. In this study, we demonstrate the level of pSTAT3 Tyr705 is increased in the hypoxic regions of human epithelial ovarian cancer (EOC) specimens, as determined by HIF-1α and CD-31 staining. *In vitro* mutagenesis studies proved that pSTAT3 Tyr705 is necessary for cell survival and proliferation under hypoxic conditions. In addition, we show that S1PR1, a regulator of STAT3 transcription via the JAK/STAT pathway, is highly expressed in hypoxic ovarian cancer cells (HOCCs). Knock down of S1PR1 in HOCCs reduced pSTAT3 Tyr705 levels and was associated with decreased cell survival. Treatment of HOCCs with the STAT3 inhibitor HO-3867 resulted in a rapid and dramatic decrease in pSTAT3 Tyr705 levels as a result of ubiquitin proteasome degradation. STAT3-target proteins Bcl-xL, cyclin D2 and VEGF showed similar decreases in HO-3867 treated cells. Taken together, these findings suggest that activation of STAT3 Tyr705 promotes cell survival and proliferation in HOCCs, and that S1PR1 is involved in the initiation of STAT3 activation. Targeting hypoxia-mediated STAT3 activation represents a therapeutic option for ovarian cancer and other solid tumors.

## INTRODUCTION

Ovarian cancer is the 5^th^ leading cause of cancer-related deaths among women and the leading cause of gynecologic cancer mortality. Despite advancements in the field of cancer therapeutics, there has been minimal improvement in survival over the past 30 years. While many women will have a response to cytoreductive surgery and adjuvant chemotherapy, the majority will recur with chemotherapeutic-resistant tumors. Further advancement demands a better understanding of the pathogenesis and genetics of this devastating disease.

Tumor hypoxia is common feature of many solid tumors. This phenomenon was first described 50 years ago when cells out of the range of oxygen diffusion were observed to necrose [1]. Since that time, hypoxia has been documented in numerous solid tumors and has been associated with multidrug resistance, invasion, metastasis, and angiogenesis [2-4]. There is evidence that ovarian cancer can be severely hypoxic, contributing to its chemotherapeutic resistance and ultimately treatment failure [5, 6]. At the cellular level, hypoxia initiates a variety of responses including activation of proto-oncogenes. Signal transducer and activator of transcription 3 (STAT3) is one such target.

Many cancer cell lines and primary tumors, including ovarian cancer, are reported to possess constitutively-active STAT3 (pSTAT3) that can lead to cellular transformation and ultimately tumorigenesis. STAT3 positivity is higher in advanced-stage disease and pSTAT3 has been correlated with disease stage, degree of differentiation, and lymph node metastasis [7, 8]. High nuclear expression of pSTAT3 has been correlated with poor overall survival and activation of STAT3 has been shown to be higher in drug-resistant recurrent tumors and resistant cell lines [9, 10].

Hypoxia (<2% O2) results in upregulation of pSTAT3 Tyr705 in human ovarian cancer cell lines and induction of resistance to both cisplatin and paclitaxel [5]. The observed resistance appears to be due to enhanced STAT3 activation. Indeed, others have shown that drug-resistant, recurrent ovarian tumors have higher pSTAT3 compared to matched primary tumors [9]. It can be hypothesized that compounds that inhibit STAT3 activation could be effective in reducing the oncogenic potential of this protein, and provide targeted therapy for hypoxic, chemotherapy-resistant ovarian cancer.

We developed a novel class of compounds that are fluoro-substituted analogs of curcumin, called diarylidenylpiperiden-4-ones (DAPs). One of these compounds, designated as HO-3867, displays selective cytotoxicity to cancer cells and has shown promise as a potential anticancer drug through the targeting STAT3 [10-14]. However, the efficacy and mechanisms whereby HO-3867 inhibition takes place under hypoxic conditions are not well known, and we investigated the targeting of pSTAT3 by HO-3867 in hypoxic tumor micro environment. Understanding what we know about hypoxia and chemotherapeutic resistance, there is an important opportunity to investigate novel therapeutic compounds for the treatment of hypoxia-mediated chemoresistance in ovarian cancer [15]. We demonstrate that advanced-stage ovarian cancers have high pSTAT3 Tyr705 expression that is focused in the hypoxic cores of tumor nodules. *In vitro* mutagenesis studies confirm that phosphorylation of STAT3 at the Tyr705 residue is necessary for cell survival and proliferation under hypoxic conditions. We furthermore describe the efficacy and mechanism of the novel anticancer agent HO-3867 under hypoxic conditions. Specifically, it targets tyrosine-phosphorylated STAT3 and consequently inhibits cancer cell proliferation and induces apoptosis via a mechanism involving ubiquitin-proteasome degradation.

## RESULTS

### Effect of hypoxia on STAT3 expression in ovarian cancer cells and hypoxic preconditioning of cancer cells on xenograft mice

**Figure 1:**
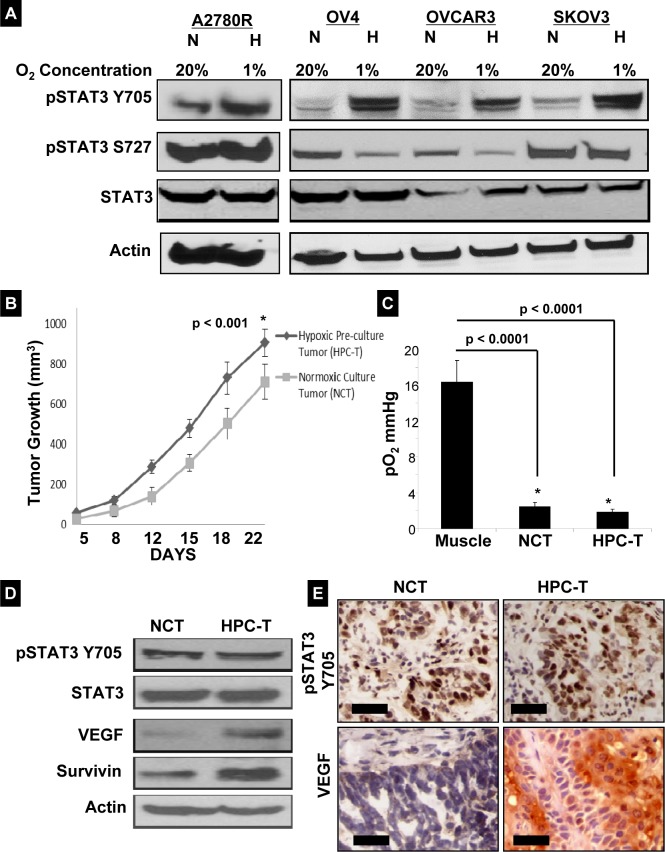
Hypoxia-induced expression of pSTAT3 Tyr705 (A) Western blot analysis of 4 ovarian cancer cell lines incubated for 24 hours under normoxic (N) (20% O_2_) or hypoxic (H) (1% O_2_) conditions. In all cell lines, pSTAT3 Tyr705 expression increased with hypoxic treatment. The response of pSTAT3 Ser727 to hypoxia was variable between cell lines; there was no change in total STAT3 levels. (B) Normoxic (NCT) and hypoxic-preconditioned (HPC-T) tumor xenografts were established in nude mice (n=4 mice per group). The tumor size was measured twice weekly with Vernier calipers in three dimensions for 3 weeks. Each data point represents mean ± SD. The HOCCs promoted faster and larger volume of tumor growth (*p < 0.001* when comparing HPC-T vs NCT at Day 22). (C) Partial pressure of oxygen (pO_2_) in murine xenograft tumors by *in vivo* EPR oximetry. Shown are pO_2_ values obtained from ovarian tumors grown by subcutaneous implantation of respective cells in xenograft mice. The results showed that the ovarian xenograft tumors are severely hypoxic when compared to muscle tissue (*p < 0.0001*). (D) Immunoblot analysis of tissue lysates of xenograft tumors. The expression of pSTAT3 Tyr705 was not significantly different between groups after 3 weeks. However, STAT3 target proteins such as VEGF and survivin were significantly higher in tumors generated from HOCCs. (E) pSTAT3 Tyr705 and VEGF expression levels confirmed by immunohistochemistry. While there appeared to be no significant difference in pSTAT3 Tyr705 expression, VEGF expression was higher in tumors originating from HOCCs, (Scale bar = 30 μm).

The effect of hypoxic culture conditions (1% O_2_) on ovarian cancer cell lines was examined. Figure 1A compares the expression of pSTAT3 Tyr705 and Ser727 under normoxic and hypoxic conditions. Hypoxia was associated with increased expression of pSTAT3 Tyr705 in all cell lines tested. In two cells lines, A2780R and SKOV3, no apparent change in pSTAT3 Ser727 expression was seen while expression was decreased in OV4 and OVCAR3 cell lines. Changes in pSTAT3 expression were not associated with altered expression of total STAT3 in any of the cell lines tested. One objective of this study was to examine the effect of hypoxic preconditioning of ovarian cancer cells on xenograft tumor growth. The ovarian cancer cell line A2780 was selected because the cells demonstrated the most notable increase in expression of pSTAT3 Tyr705 in response to hypoxia *in vitro*. BALB/c mice were injected with either A2780 cells grown under normoxic (20% O2) or hypoxic (1% O_2_) conditions for 3 weeks. Tumors of HOCC-injected mice (HPC-T) were larger and grew more rapidly than xenograft tumors of cells cultured under normoxia (NCT) (Fig. 1B). EPR oximetry was used to determine tumor oxygen levels. Figure 1C shows that both ovarian tumor xenografts were severely hypoxic (<2 mmHg) when compared to gastrocnemius muscle tissue (≅15.16 mmHg). Tumor tissues extracted from mice showed consistent expression of pSTAT3 Tyr705 in both types of tumor. There was a difference in pSTAT3 Tyr705 expression between the two groups after 2 weeks, which was no longer evident by the 3^rd^ week (*data not shown*). Interestingly, expression of proteins downstream of STAT3, including VEGF and survivin, was higher in tumors of HOCC-injected mice after 3 weeks (Fig. 1D). These findings were confirmed via IHC performed on tumor specimens (Fig. 1E). Based on these findings, there is a clear distinction between tumors derived from HOCCs as compared to those developed from normoxic cancer cells. Hypoxic preconditioning of ovarian cancer cells results in xenograft tumors that display enhanced tumor growth that is at least mediated in part via activation of the STAT3 signaling pathway.

### Expression of pSTAT3 Tyr705 in advanced ovarian tumor

**Figure 2:**
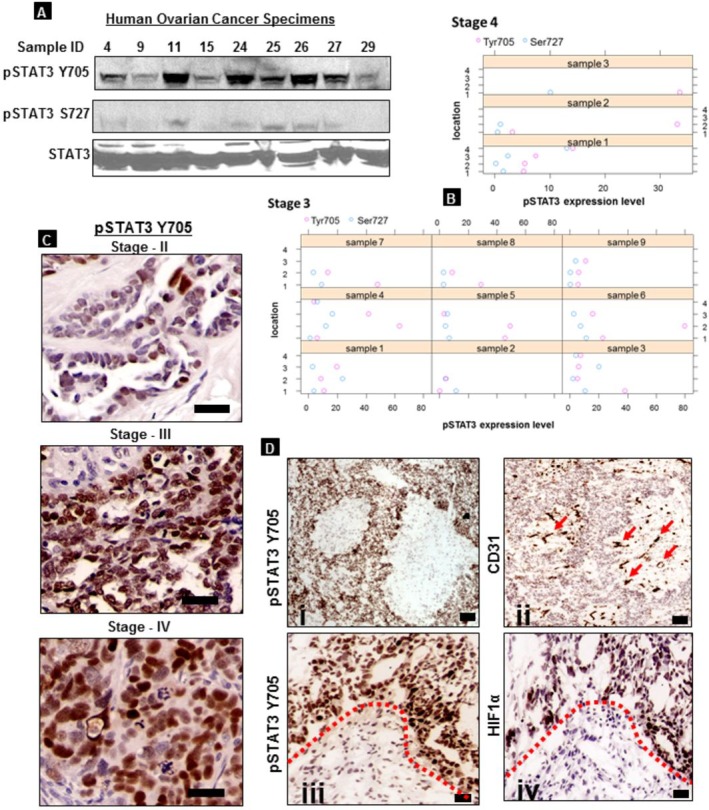
pSTAT3 Tyr705 and Ser727 expression in advanced human ovarian tumors (A) Representative immunoblot of human ovarian cancer specimens shows increased expression of pSTAT3 Tyr705 compared to Ser727 in all samples. (B) Dot plots showing densitometric expression of pSTAT3 Tyr705 and Ser727 in the human samples tested (Stage IV n=3; Stage III n=9) versus location of extraction. pSTAT3 Tyr705 expression was significantly higher than pSTAT3 Ser727 in stage III (p=0.0025) and stage IV (p=0.0156) disease. (C) Immunohistochemical analysis of stage II, III and IV human ovarian cancer specimens compared with early stage II, showing increased expression of pSTAT3 Tyr705 in advanced ovarian tumors. (D) pSTAT3 (Y705) expression in hypoxic regions of human ovarian high-grade serous carcinomas. The pattern of expression of pSTAT3 Tyr705 in advanced-stage human ovarian high grade serous carcinomas was analyzed on consecutive sections using immunohistochemistry. High levels of pSTAT3 expression (i, iii) were observed in hypoxic areas identified by their distance from CD31-positive microvasculature (arrows in image ii) as well as expression of HIF1α (iii). The dotted line separates pSTAT3 positive area from the adjacent region in image iv. (Scale bar = 100 μm)

Analysis of STAT3 expression in human ovarian cancer specimens was examined via western blot and immunohistochemistry. Figure 2A shows a representative immunoblot of human tumor samples of women with advanced-stage disease showing higher expression of pSTAT3 Tyr705 as compared to pSTAT3 Ser727. The dot plots in Figure 2B are depictions of the average levels of pSTAT3 Tyr705 expression, grouped by grade (Stage III or Stage IV) and location from which the tumor was extracted in the 33 human samples tested. There was a statistically-significant difference in expression of Tyr705 compared to Ser727 in stage III (p=0.002) and not in stage IV (p=0.06) disease. IHC showed higher pSTAT3 (Tyr705) expression in advanced-stage ovarian cancer specimens (stages III and IV) when compared with stage II (Figure 2C). In addition, we examined whether activated STAT3 expression is increased in hypoxic regions of human tumor samples. Figure 2D shows the pattern of expression of pSTAT3 Tyr705 in ovarian tumors analyzed in advanced stages of tumor growth using immunohistochemistry on consecutive sections. High levels of pSTAT3 expression (images i and iii) were observed in hypoxic areas of the tumor, identified by their distance from the terminal ends of CD31-positive vasculature (image ii, identified by red arrow symbols). This was further confirmed by expression of hypoxic marker HIF-1α in the cells (iv) located in tumor regions corresponding to the areas expressing high levels of pSTAT3 (iii). Taken together, our results suggest pSTAT3 Tyr705 is highly expressed in ovarian cancer cells grown under hypoxic conditions *in vitro* as well as in human ovarian epithelial cancer specimens within areas of necrosis/hypoxia.

### Effect of deletion mutant at Tyr705 in ovarian cancer cells

**Figure 3:**
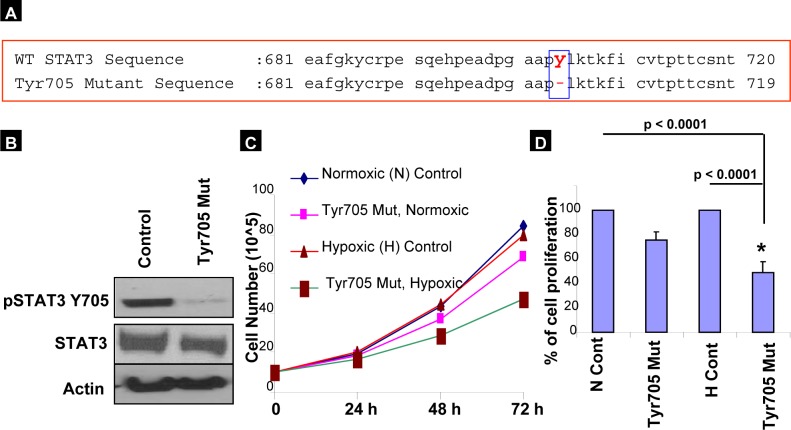
STAT3 Tyr705 activation is important for cell proliferation in hypoxic ovarian cancer cells (A) sequence of STAT3 gene segment (681 to 720), with the deletion mutant at Y705 outlined. (B) The deletion mutants were transfected into A2780 cells for 24 hours, and Tyr705 phosphorylation was analyzed by western blot. (C&D) The deleted mutant gene was transfected into A2780 cells and incubated both normoxic or hypoxic conditions. Cell proliferation and survival were similar among wild-type cells grown under both normoxic and hypoxic conditions, however both were decreased among cells transfected with mutant Tyr705. This was most pronounced among those cells grown under hypoxic conditions (mutant Tyr705 cell proliferation vs Normoxic control *p-value < 0.0001*; vs Hypoxic control *p-value < 0.0001*).

We next investigated the role of the specific activation of STAT3 Tyr705 in hypoxic ovarian cancer cells using a deletion mutant at Tyr705 (Phusion Site-Directed Mutagenesis method, Thermo Scientific). Figure 3A shows the sequence of wild-type STAT3 gene segment (681 to 720), and the corresponding mutant in which the deletion at Y705 is outlined. The deleted mutant of STAT3 Tyr705 was transfected into A2780 cells, which was confirmed by western blotting demonstrating low tyrosine phosphorylation in the transfected A2780 cells compared with un-transfected control cells (Figure 3B). Further, we evaluated the role of Tyr705 under hypoxic and normoxic conditions. The deleted mutant gene was transfected into A2780 cells, which were subsequently incubated under normoxic or hypoxic conditions, and examined for cell survival and proliferation. Our results show that the specific deletion of STAT3 Tyr705 suppresses cell survival and cell proliferation (53 to 67%) when compared with both un-transfected and normoxic cells transfected with the deletion mutant gene (Figures 3C & 3D). Our previous study showed that silencing of STAT3 by STAT3 siRNA followed by treatment with cisplatin or taxol under hypoxic conditions showed a significantly decreased (>70%) cell viability [5]. This suggests that specific activation of Tyr705 is required for cell proliferation, survival, and possibly chemoresistance under hypoxic conditions.

### S1PR1 regulates STAT3 expression in hypoxic ovarian cancer cells

**Figure 4:**
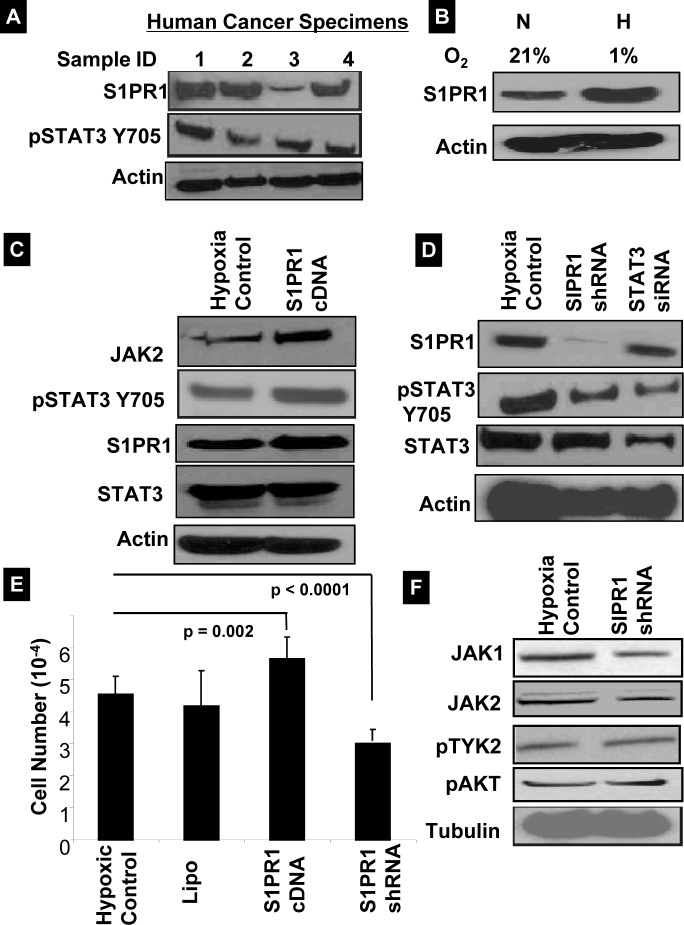
S1PR1 expression in HOCCs induces STAT3 activation (A) Western blot obtained from four human ovarian cancer specimens demonstrated consistent expression of S1PR1 and pSTAT3 Tyr705. (B) A2780 cell line incubated for 24 hours under hypoxic (H) conditions demonstrated increased expression of S1PR1 as compared to normoxia (N). (C) A2780 cells were transfected with S1PR1 cDNA gene under hypoxic conditions and subjected to western blot analysis after 24 hours. The S1PR1-transfected cells had increased pJAK2 and pSTAT3 Tyr705 expression levels. (D) S1PR1 shRNA and STAT3 siRNA were transfected into A2780 cells under hypoxic conditions. After 24 h, cells were collected and analyzed by western blot. Results showed that silencing the S1PR1 gene was associated with decreased pSTAT3 expression; conversely, STAT3 siRNA showed a non-significant decrease in S1PR1 levels. (E) MTT cell viability studies after transfection with S1PR1 cDNA and shRNA into A2780 cells under hypoxic conditions. S1PR1 cDNA increased cell viability Cell survival (*p = 0.002* vs control) while S1PR1 shRNA resulted in decreased cell viability (*p < 0.0001* vs control). (F) S1PR1 shRNA decreased JAK1 and pJAK2 levels without any changes of pTYK2, and pAkt.

Recent evidence suggests that S1PR1 induces STAT3 activity in cancer cells [19, 20]. Thus, we sought to examine the expression of S1PR1 in human ovarian cancer, and found that S1PR1 is consistently expressed in human ovarian cancer samples (Fig. 4A). We then examined the activity of S1PR1 under hypoxic conditions as a possible mediator of the increased expression of pSTAT3 seen in HOCCs. Indeed, S1PR1 expression was found to be significantly higher in HOCCs (Fig. 4B). Furthermore, the overexpression of S1PR1 with cDNA increased pSTAT3 expression via activation of JAK2 (Fig. 4C). To examine the effect of S1PR1 inhibition on STAT3, HOCCs were transfected with S1PR1 shRNA. This resulted in the inhibition of pSTAT3 Tyr705 and decreased cell survival (Fig. 4D & E). Interestingly, total STAT3 expression was not affected by inhibition of S1PR1 (Fig. 4D). This supports previous observations and suggests that S1PR1-induced expression of STAT3 is part of a positive feedback loop resulting in persistent STAT3 activation in ovarian cancer. Furthermore, suppression of S1PR1 was associated with decreased JAK1 and JAK2 expression but no changes in other STAT3-regulating kinase pathways such as TYK2 or AKT (Fig. 4F). Our results suggest that S1PR1 induces STAT3 activation and is involved in a positive feedback loop through the activation of JAK signaling pathways in HOCCs.

### HO-3867 targets pSTAT3 Tyr705 in hypoxic ovarian cancer cells

**Figure 5:**
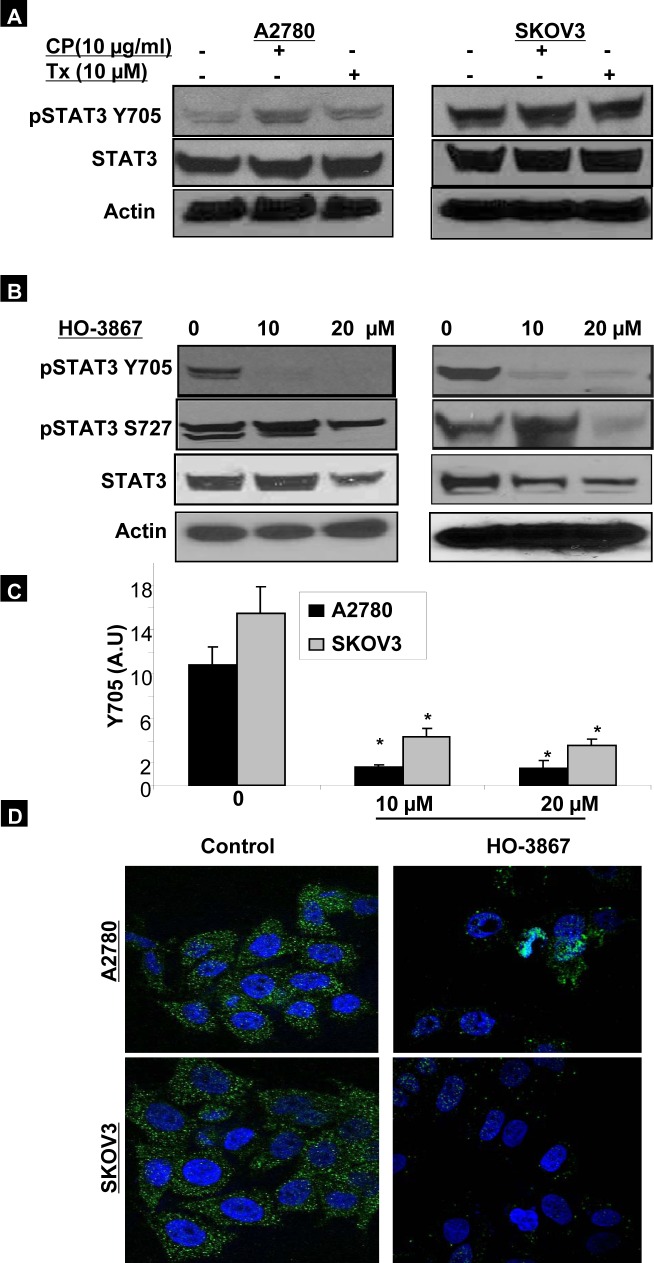
HO-3867 targets pSTAT3 Tyr705 A2780 and SKOV3 cells were cultured under hypoxic conditions (1% O_2_) and treated with cisplatin 10 g/mL, paclitaxel 10 μM, or HO-3867 for 2 hours. After treatment, cells were subject to western blot analysis and immunocytochemistry. (A) Treatment with cisplatin resulted in slight upregulation of pSTAT3 Tyr705 in A2780 cells, but had no effect in SKOV3 cells. Paclitaxel had no effect on pSTAT3 expression in either cell line. (B) Treatment with HO-3867 for only 2 hours under hypoxic conditions resulted in decreased expression of pSTAT3 Tyr705 and pSTAT3 Ser727 at doses as low as 10 μM. (C) Densitometric analyses of visualized bands for pSTAT3 Tyr705 expression. Data represent mean ± S.E. of three independent experiments. *, *p*<0.05 as compared with control group. (D) Immunocytochemical analysis revealed that treatment with HO-3867 resulted in decreased cytoplasmic and nuclear expression of pSTAT3 Tyr705.

We have previously reported that cisplatin and paclitaxel are far less effective in eliminating hypoxic ovarian cancer cells [5]. Here we examined the effect of HO-3867 on pSTAT3 expression as compared to cisplatin and paclitaxel. In Figure 5A, A2780 and SKOV3 cells were cultured in hypoxic (1% O_2_) conditions for 24 hours and then subjected to two-hour treatment with cisplatin at 10 g/mL or paclitaxel at 10 μM. Treatment with cisplatin increased the expression of pSTAT3 Tyr705 in A2780 cells but had no effect on expression in SKOV3 cells. Paclitaxel had no effect on pSTAT3 Tyr705 expression in either cell line. It is possible that hypoxic activation of STAT3 might mediate cellular resistance to chemotherapy. Our previous reports have shown that our novel synthetic compound, HO-3867, works, at least in part, via inhibition of the STAT3 signaling pathway in various cancer cell lines even at very early time points (10). We therefore examined the effects of HO-3867 on HOCCs (Fig. 5B). Within only 2 hours, treatment with either 10 μM or 20 μM of HO-3867 resulted in significantly decreased expression of pSTAT3 Tyr705. Figure 5C depicts the densitometric analyses of visualized bands for pSTAT3 Tyr705 expression. In addition, we confirmed the nuclear expression of Tyr705 by IHC analysis as shown in Figure 5D. After 2 hours of treatment with 20 μM HO-3867 there was drastically-decreased nuclear and cytoplasmic expression of pSTAT3 Tyr705 in both A2780 and SKOV3 cells.

### HO-3867 promotes proteasome-ubiquitin degradation of Tyr705-phosphorylated STAT3

**Figure 6:**
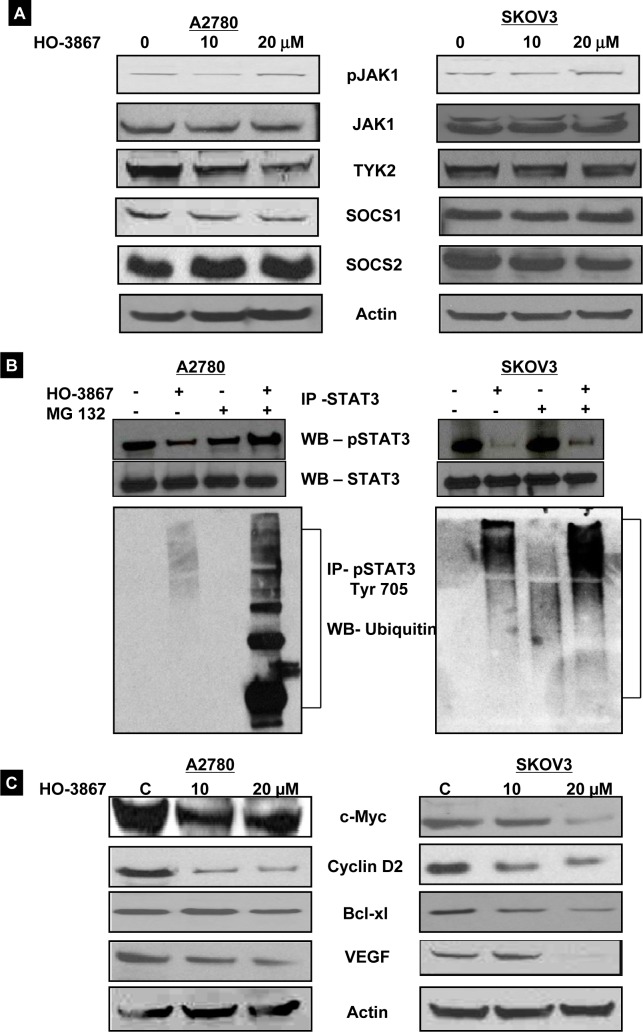
HO-3867 targets pSTAT3 Tyr705 specifically via ubiquitin-proteosome degradation A2780 and SKOV3 cells were cultured under hypoxic (1% O_2_) conditions and subsequently treated with 10 μM or 20 μM of HO-3867 for 2 hours. Cells lysates were analyzed via western blot. (A) HO-3867 did not alter expression of upstream proteins including TYK2/pTYK2, JAK1/pJAK1, or SOCS 1 and 2. (B) The intense smear of poly-ubiquitinated pSTAT3 Tyr705 after treatment with HO-3867 and the proteosome inhibitor MG132 indicates that HO-3867 targets STAT3 via initiation of proteosomal degradation. Cell lysates were immunoprecipitated with STAT3 or pSTAT3 Tyr705 antibody. The precipitates were subjected to SDS-PAGE and immunoblotting using STAT3, pSTAT3 Tyr705, and ubiquitin antibodies. (C). STAT3-target protein expression is decreased after treatment with HO-3867. A2780 and SKOV3 cells were cultured under hypoxic (1% O_2_) conditions and subsequently treated with 10 μM or 20 μM HO-3867 for 2 hours. Cells lysates were analyzed via western blot. Expression of anti-apoptotic protein Bcl-xL, pro-survival protein c-myc as well as cell-cycle-related proteins including cyclin D2 were decreased following HO-3867 treatment.

There are reports of STAT-family proteins being regulated by ubiquitin-mediated degradation [17, 21, 22]. We sought to determine if proteasomal degradation was responsible for HO-3867's effect on pSTAT3 Tyr705. First, western blot analysis was used to evaluate the STAT3-regulating upstream pathways. A2780 and SKOV3 cells were cultured under hypoxic conditions and treated with HO-3867 for 2 hours. Figure 6A shows that treatment with HO-3867 had no effect on upstream protein expression, including the positive regulators pJAK1/ JAK1, pTYK2/TYK2 and negative regulators SOCS1 and SOCS2. HO-3867 also does not appear to exert its effects via interference with the positive feedback loop between S1PR1 and STAT3. Our previous study was reported that HO-3867 inhibits pJAK1 in normoxic condition cells were treated with 24 hours [11]. Under hypoxic conditions, HO-3867 did not alter the Jak pathways within 2 hours of treatment, while inhibiting pSTAT3. Next, we investigated the possibility of ubiquitin-mediated degradation. A2780 and SKOV3 cells were cultured under hypoxic conditions and treated with a combination of MG132, a proteasome inhibitor, and HO-3867. Figure 6B shows the results of immunoprecipitation and western blot analysis. The intense smear of poly-ubiquitinated pSTAT3 Tyr705 proteins in both cells lines treated with MG132 and HO-3867 suggests that HO-3867 preferentially promotes the ubiquitin-proteasome degradation of pSTAT3 Tyr705 in human HOCCs. While there was no change in expression of upstream regulatory proteins, downstream target proteins of STAT3 were accordingly decreased after treatment with HO-3867 in both cell lines cultured under hypoxic conditions. Figure 6C shows the downstream proteins upregulated by STAT3 activation, including those involved in cell proliferation (c-Myc, and cyclin D2), anti-apoptosis (Bcl-xl), and angiogenesis (VEGF). The downregulation of these proteins suggests the profound antitumor potential of HO-3867 by inhibition of STAT3 activation and its target proteins, even in hypoxic cancer cells.

### HO-3867 inhibits cell proliferation and induces apoptosis

**Figure 7:**
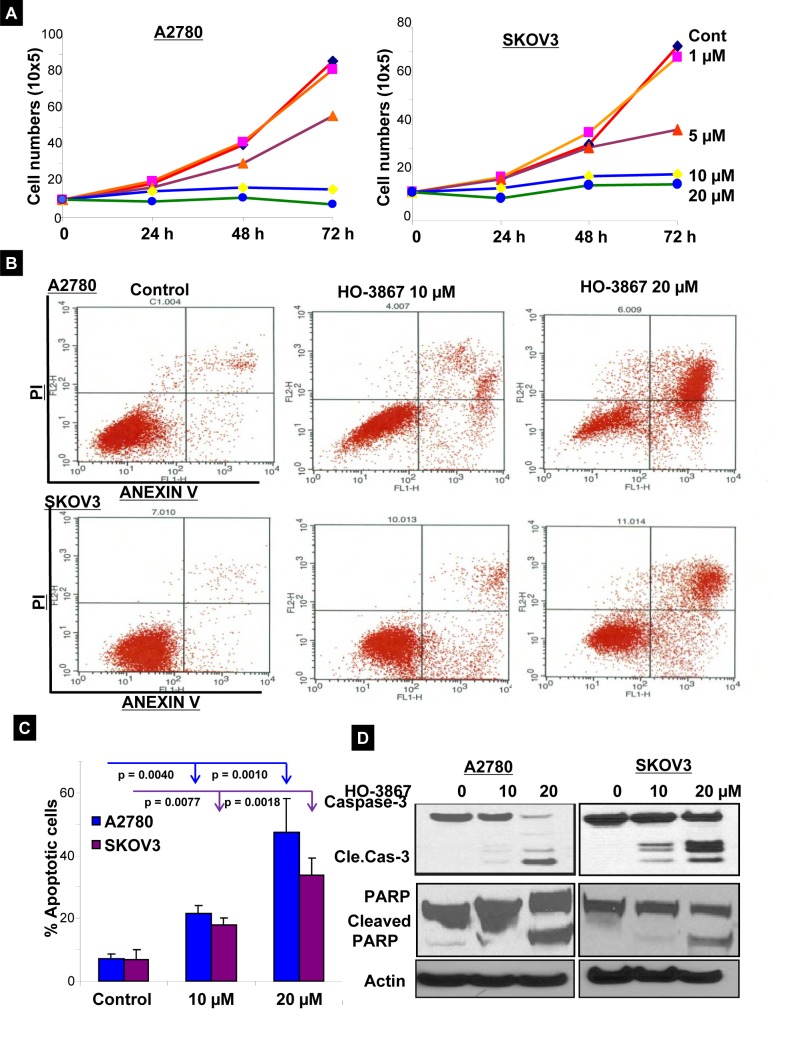
HO-3867 inhibits cell proliferation and induces apoptosis (A) A2780 (left panel) and SKOV3 (right panel) cells were cultured under hypoxic conditions (1% O_2_) for 24 hours. They were subsequently treated with various concentrations HO-3867 for 24, 48, or 72 hours and counted. The graphs display dose-dependent inhibition of cell proliferation induced by HO-3867. (B) Treatment of A2780 and SKOV3 cells with 10 μM and 20 μM of HO-3867 for 24 hours followed by Annexin V analysis showed a dose-dependent increase in apoptosis. (C) Graphical representation of flow cytometry results displays a significant increase in the percentage of apoptotic cells with increasing concentrations of HO-3867. (D) Representative western blot analysis shows a dose-dependent increase in cleaved-caspase-3 and cleaved PARP after treatment with HO-3867.

Next we assessed whether the inhibition of STAT3 and its target proteins resulted in cell growth arrest and apoptosis in HOCCs. Figure 7A is a graphical representation of cell counting assays performed on A2780 and SKOV3 cells cultured under hypoxic conditions. Cells were treated with increasing concentrations of HO-3867 over the course of 72 hours and counted. The graphs show that HO-3867 inhibits proliferation of hypoxic ovarian cancer cells in a dose-dependent manner. We then evaluated whether HO-3867 induced apoptosis under hypoxic conditions. Figures 7B and 7C show the results of annexin V flow cytometry. Treatment of HOCCs with HO-3867 resulted in a dose-dependent increase in the percentage of cells in early and late apoptosis. Figure 7D shows that treatment with HO-3867 under hypoxic conditions results in increased expression of apoptotic markers cleaved-caspase-3 and cleaved PARP. These results and the findings in our previous work [11, 13, 23)] strongly suggest that HO-3867, inhibits cell growth and induces apoptosis under conditions of normoxia (20% O_2_) or hypoxia (1% O_2_).

## DISCUSSION

Hypoxia is one of the factors that have been shown to increase the oncogenic activation of STAT3. Although it is known that the constitutively-active form of STAT3 plays key roles in tumorigenesis and tumor progression, the molecular mechanism by which the STAT3 is activated in hypoxic cancer cells is poorly understood. In addition, there is no evidence that commercially-available STAT3 inhibitors are effective in treating hypoxic cancer cells. In this study, we show that HO-3867, a novel curcumin analogue, targets hypoxia-stimulated pSTAT3 Tyr705, at least in part, via the ubiquitin-proteasome degradation pathway, resulting in apoptosis and downregulation of proteins involved in cell survival, proliferation, and angiogenesis.

In the current study, for the first time, we show increased activation of STAT3 in hypoxic regions of human epithelial ovarian cancer specimens. This result is important when one considers the association of pSTAT3 expression with chemotherapeutic resistance in ovarian, hepatocellular, and head and neck cancers [5, 24]. In the current study, we show that treatment with cisplatin under hypoxic conditions results in increased pSTAT3 Tyr705 expression. It is possible that this increased pSTAT3 expression contributes to development of cisplatin resistance in ovarian cancer cells. To our knowledge, this is also the first study to suggest that culturing ovarian cancer cells in hypoxic conditions promotes faster tumor growth in an *in vivo* animal model after hypoxic preconditioning by increasing the expression of STAT3 target genes in ovarian cancer cells and xenograft tumors.

Although several studies have identified STAT3 as a focus of therapeutic intervention [11, 13, 17, 25-30], the specific targeting of STAT3 Tyr705 in hypoxic ovarian cancer has not been investigated. Our current data shows that tyrosine-phosphorylated STAT3 is highly expressed in advanced ovarian cancer and suggests that pSTAT3 Tyr705 may specifically be activated in those ovarian cancers that are hypoxic or have regions of hypoxia. Tyrosine phosphorylation is the most important mediator of STAT3's function as an oncogene [31]. In the current study, we have proved that the specific activation of STAT3 Tyr705 is required for cell proliferation and survival in hypoxic ovarian cancer.

The increased expression of pSTAT3 in HOCCs appears to be mediated by S1PR1. Our findings show that S1PR1 induces STAT3 activation, at least in part, through the JAK2 tyrosine kinase. Specifically, we demonstrate that increasing S1PR1 expression in HOCCs initiates STAT3 activation, resulting in enhanced ovarian cancer cell survival and proliferation under hypoxic conditions both *in vitro* and *in vivo*. Other investigators have demonstrated that S1PR1 is capable of activating STAT3 through the JAK2 tyrosine kinase, providing confirmation of our findings [19]. Whether STAT3 activity regulates S1PR1 in HOCCs via a positive feedback loop remains to be determined.

Knowing that the therapeutic efficacy of many agents is limited under hypoxic conditions, HO-3867 presents itself as an agent with promise that is active under hypoxic conditions. The compound targets one of the main mediators of oncogenesis and therapeutic resistance, STAT3 activation. Curcumin is known to have anti-tumor effects via the STAT3 signaling pathway; however its use is limited by its poor bioavailability. Other curcumin analogues mediate their effects via targeting upstream proteins such as the JAK kinases [32-34]. We have shown that HO-3867 has anticancer properties under hypoxic conditions with no effect on upstream mediators of STAT3 activation. Instead, this compound specifically promotes pSTAT3 Tyr705 degradation via the ubiquitin-proteasome pathway. While there have been other reports of ubiquitin-mediated degradation of STAT3 [17, 21, 30], this is the first report of tyrosine-phosphorylated STAT3 being regulated by this pathway in hypoxic cancer cells. This novel effect of HO-3867 may provide insight into the antitumor properties of curcumin analogs as a group or may be specific to the hypoxic microenvironment, but requires further investigation.

In conclusion, pSTAT3 Tyr705 is highly expressed by HOCCs as well as in areas of necrosis and presumed our findings provide additional evidence of S1PR1's involvement in STAT3 activation and, for the first time, demonstrate this effect in hypoxic cancer cells and tumors. Knowing that the hypoxic microenvironment fosters malignant cell proliferation and resistance to chemotherapy, targeting hypoxia–mediated tyrosine phosphorylation with the novel anticancer function of HO-3867 has a potential therapeutic advantage of overcoming hypoxia-induced chemo-therapeutic resistance in ovarian cancer.

## METHODS

### Materials

HO-3867 was synthesized in the laboratory [16]. Stock solutions of the compound were freshly prepared in dimethylsulfoxide (DMSO). Cell-culture medium (RPMI 1640 and DMEM), fetal bovine serum (FBS), antibiotics, sodium pyruvate, trypsin, and phosphate-buffered saline (PBS) were purchased from Gibco (Grand Island, NY). Polyvinylidene fluoride (PVDF) membrane and molecular-weight markers were obtained from Bio-Rad (Hercules, CA). Antibodies against pSTAT3 Tyr705, pSTAT3 Ser727, pJak1, Jak1, pTyk2, Bcl-2, actin, caspase-3, and PARP were purchased from Cell Signaling Technology (Beverly, MA). Antibodies specific for STAT3, SOCS1, SOCS2, cyclin D2, Bcl-2, VEGF, p53, and ubiquitin were purchased from Santa Cruz Biotechnology (Santa Cruz, CA). Enhanced chemiluminescence (ECL) reagents were obtained from Amersham Pharmacia Biotech (GE Healthcare, Piscataway, NJ). All other reagents, of analytical grade or higher, were purchased from Sigma-Aldrich.

### Patient Samples

Solid tumor samples from 33 patients with primary epithelial ovarian cancer or benign ovarian neoplasms that had undergone initial surgery at The Ohio State University Medical Center were obtained. Samples were homogenized in non-denaturing lysis buffer and subject to western blot analysis as described earlier. The use of human tissues in this study was approved by the Institutional Review Board of The Ohio State University Wexner Medical Center.

### Cell lines and culture

A2780, A2780R, SKOV3, OV4, and OVCAR3 human epithelial ovarian cancer cell lines were used in this study. The cells were grown in RPMI 1640 or DMEM medium supplemented with 10% FBS, 2% sodium pyruvate, 1% penicillin and 1% streptomycin. Cells were grown in a 75 mm flask to 70% confluence at 37° C in an atmosphere of 5% CO_2_ and 95% air. Hypoxic exposure was achieved by incubating cells in a humidified atmosphere of 1% O_2_ and 5% CO_2_ in a specialized environmental chamber (C-Chamber and ProOx Model C21, Biospherix, USA) contained within a standard cell culture incubator. Cells were routinely trypsinized (0.05% trypsin/EDTA) and counted using an automated counter (NucleoCounter, New Brunswick Scientific, Edison, NJ).

### Cell Proliferation and Apoptosis

A2780 and SKOV3 cells were cultured in RPMI and DMEM medium, respectively. They were seeded into 60 mm culture dishes and cultured for 24 hours, followed by treatment with varying concentrations (1, 5, 10, and 20 μM) of HO-3867 and counted using a NucleoCounter (New Brunswick Scientific, Edison, NJ) after 24, 48, or 72 hours of treatment. A2780 and SKOV3 cells were treated with 10 μM and 20 μM of HO-3867 for 24 hours in hypoxic culture conditions. Apoptotic cells were measured by flow cytometry using Annexin V.

### Immunoblot analysis

Cells in RPMI 1640 or DMEM medium were treated with DMSO (control) or HO-3867 (10 μM or 20 μM) for 2 or 24 hours. Equal volumes of DMSO (0.1% v/v) were present in each treatment. Following treatment, the cell lysates were prepared in non-denaturing lysis buffer as previously described [12].

### Site-directed Mutagenesis

Phusion Site-Directed Mutagenesis (Thermo Scientific) was used to create STAT3 Tyr705 deletion mutants. Primers used create the STAT3 Y705 deletion mutation are: 5′ CTGAAGACCAAGTTTATCTGTGTG ACACCAAC and 5′ TGGGGCAGCGCTACCTGG.

### siRNA and cDNA Study

A2780R cells were transfected with STAT3 siRNA (Santa Cruz-sc-29493) or SIPR1 shRNA (AAAGAGACGCTTTCACATGGG) and negative control siRNA using Lipofectamine 2000 (Invitrogen) according to the manufacturer's instructions. The cell lysates were subjected to immunoblot analysis as described above for Jak1, Jak2, pSTAT3 and SIPR1. S1PR1 overexpression experiments were performed using a wild-type cDNA. Cells were subjected to immunoblot analysis with pSTAT3 Tyr705 and STAT3 antibodies 24 hours after transfection of the S1PR1 gene.

### Immunocytochemistry

Cells in RPMI or DMEM medium were seeded onto sterile glass coverslips in 6-well plates with an average population of 50,000 cells/well. After 24 hours of culture in hypoxia (1% O_2_), cells were treated with 10 μM or 20 μM HO-3867 for 2 hours. The cells were then washed, fixed, and incubated with primary antibody according to a previously described protocol [17].

### Immunoprecipitation

Cells in RPMI or DMEM medium were cultured under hypoxic conditions for 24 hours and subsequently treated with 20 μM HO-3867 alone or in combination with 25 μM of the proteasome inhibitor MG132 (Calbiochem) for 2 hours. Cell lysates were incubated with primary antibody and immunoprecipitated with rProtein G Agarose beads according to a previously described protocol [17].

### Ovarian tumor xenografts in mice and tumor pO2 measurement

A2780 cells were cultured and incubated in either normoxic (20% O_2_) or hypoxic (1% O_2_) conditions for 3 weeks. Cells were routinely trypsinized and passaged every week to maintain viability. Cells from either group were injected into the subcutaneous tissue on the back of 6-week-old BALB/c nude mice from the National Cancer Institute. Three to 5 days after injection, when the tumors reached 3 to 5 mm in diameter, the mice from each group were divided (n = 4/group) in a manner to equalize the mean tumor diameter among the groups. The size of the tumor was measured twice weekly using a digital Vernier caliper. The tumor volume was determined from the orthogonal dimensions (d1, d2, d3) using the formula (d1 × d2 × d3) × π/6. The tumors were then homogenized and subjected to immunoblot analysis as described above or 2.12 immunohistochemistry analysis as described below. The partial pressure of oxygen (pO_2_) within the xenograft tumor tissues grown from A2780 cells cultured under normoxic or hypoxic conditions were measured by electron paramagnetic resonance (EPR) oximetry as previously described [5]. Lithium octa-*n*-butoxy-naphthalocyanine (LiNc-BuO) probe crystals were directly implanted into the tumor or into the gastrocnemius muscle of control (non-tumor) mice.

### Immunohistochemistry

Human ovarian tumor tissues were embedded in OCT medium (Tissue Tek 4583) and stored at –70° C until sectioning. Consecutive, 5 μm tissue sections were obtained for haematoxylin and eosin (H&E) and immunohistochemical (IHC) staining, following previously-described methods.[18]

### Statistical Analysis

Results were expressed as mean ± S.E. To compare two independent treatments for continuous endpoints, the Student's t-test was used, with the assumption of unequal variances where appropriate. One-way ANOVA was used for multiple pair-wise comparisons among three or more groups. For human tissue samples, linear mixed models were used to account for the covariance structure due to the repeated measures from the same patient. P values were adjusted for multiple comparisons by Holms' procedure. A P value 0.05 or less is considered statistically significant. For the comparison between STAT3 Tyr705 and Ser727 proteins using 33 human tissue samples obtained from 12 patients (n=26 samples for stage III), we can have 80% power to detect the effect size of 0.7 from the collected data (α=0.05). The sample size for stage IV (n=7) did not provide enough power to detect the effect size of 1 as shown by the data, which implies that the analysis was exploratory.
